# Environmental Exposures and Stroke Risk: Converging Pathways, Unequal Burdens, and Prevention Opportunities

**DOI:** 10.1007/s11910-026-01505-2

**Published:** 2026-07-22

**Authors:** Muaz Ali, Whitney Mayberry, Rebecca Achey, Jukka Putaala, Amre Nouh

**Affiliations:** 1 Department of Neurology, Cleveland Clinic Florida, Weston, FL USA; 2 Department of Neurosurgery, Cleveland Clinic Foundation, Cleveland, OH USA; 3 Department of Neurology, University of Helsinki and Helsinki University Hospital, Helsinki, Finland; 4 Department of Neurology, Cleveland Clinic Foundation, Cleveland, OH USA; 5 Cleveland Clinic Department of Neurology, Cleveland Clinic Foundation, 9500 Euclid Ave, Cleveland, OH 44195 USA

**Keywords:** Stroke, Environmental exposures, Air pollution, Climate change, Social determinants of health, Exposome

## Abstract

**Purpose of Review:**

To synthesize recent evidence on how environmental exposures influence stroke risk, with emphasis on air pollution, thermal stress, circadian and occupational disruption, built and social environments, and selected toxicants.

**Recent Findings:**

Long-term and episodic air pollution, especially fine particulate matter with aerodynamic diameter ≤ 2.5 μm (PM₂.₅) and wildfire smoke, remain most consistently associated with stroke risk. Extreme heat combined with heat-humidity metrics is emerging as an important acute stroke trigger. Noise, artificial light at night, shift work, and long working hours show smaller but plausible associations, while greener and more walkable environments may be modestly protective. Social and structural disadvantage clusters, multiple exposures and inequities play an integrated role in risk and prevention.

**Summary:**

Environmental determinants are increasingly relevant to stroke risk. The strongest current links are realted to pollution, heat, sleep, and work disruption, as well as environmental inequity. Future research should move beyond single-exposure models toward combined-exposure as well as intervention-focused approaches.

## Introduction

Stroke risk is shaped not only by individual clinical risk factors, but broader social and environmental contexts. This interplay is most evident with lower socioeconomic status association with higher stroke risk, disparities in stroke care, and worse outcomes [1]. Furthermore, greater environmental variance is associated with higher population-level stroke prevalence and a higher burden of adverse cardiovascular conditions and risk factors [2]. Recent epidemiologic evidence links environmental exposures—including ambient air pollution, fine particulate matter with aerodynamic diameter ≤ 2.5 μm (PM₂.₅), wildfire-smoke PM₂.₅, nonoptimal temperature, and road-traffic noise—with stroke-related outcomes such as hospital admission, incidence, mortality, and population burden [3–10]. Among these exposures, the data is most extensive for air pollution and temperature-related thermal stress. Ambient and wildfire-smoke PM₂.₅ have been associated with increased stroke risk and burden [3–5]. Both heat and cold contribute to stroke outcomes with low temperature accounting for most temperature-attributable stroke burden worldwide and heat-related burden rising rapidly [7–10]. Road-traffic noise has also been associated with incident stroke, and multi-exposure studies suggest these environmental hazards may cluster rather than occur in isolation [3–6, 8, 9].

Additional evidence suggests that circadian disruption, including shift/night work and artificial light at night and occupational stressors such as long working hours and job strain may contribute to stroke risk, stroke-attributable burden, or stroke-related healthcare demand. Although this evidence is generally more limited and heterogeneous than the evidence for air pollution and temperature-related exposures [11–14]. Other toxicants such as arsenic, and extreme-weather disasters have also been linked [15, 16].

**Fig. 1:**
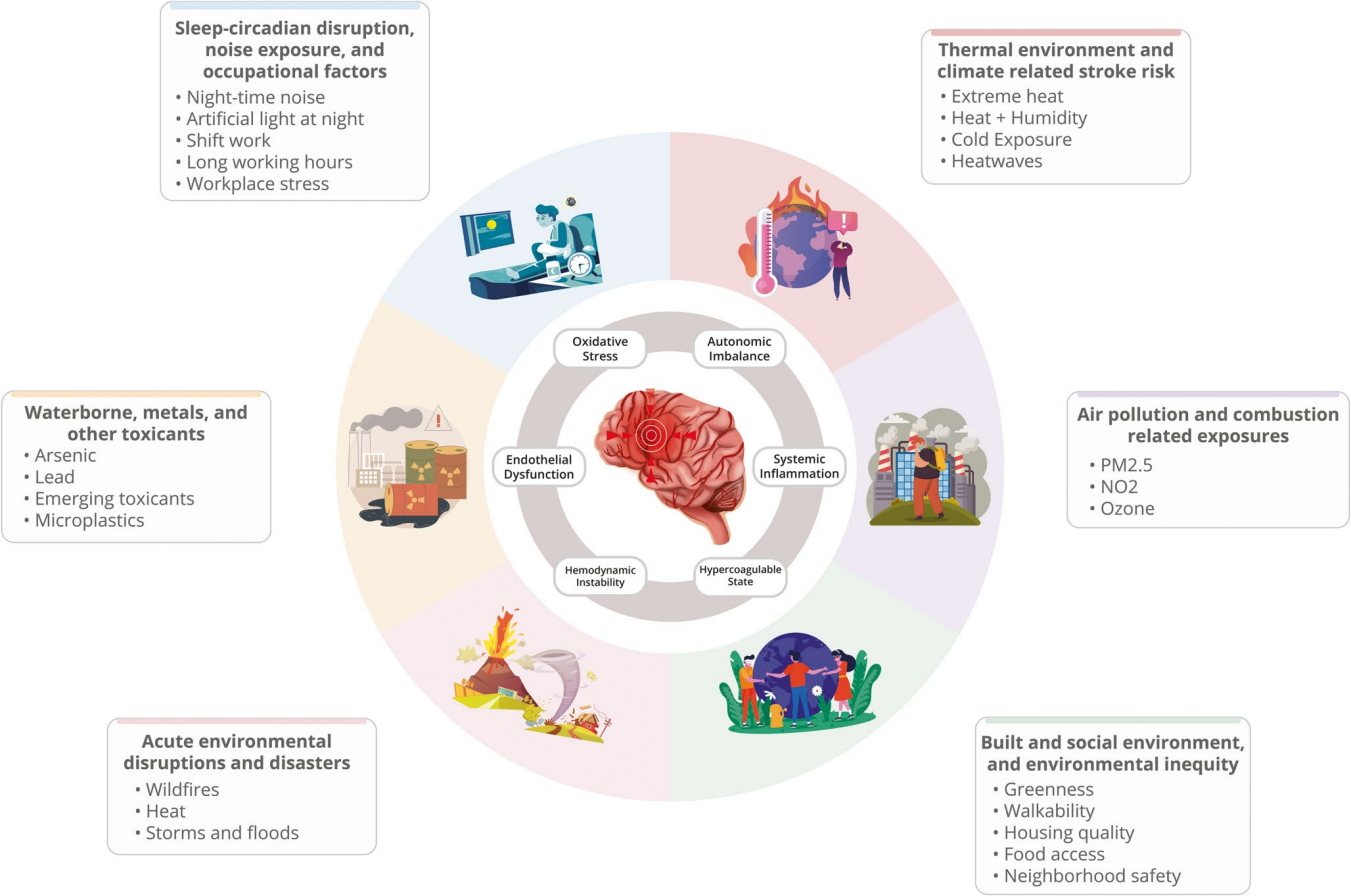
Environmental Exposures Shape Stroke Risk Through Converging Biologic and Contextual Pathways. This central illustration is a conceptual summary of major environmental domains that may influence stroke risk and is not intended to be exhaustive. Exposure categories are not mutually exclusive and may co-occur within individuals and communities. “Biologic pathways” refer to physiologic mechanisms such as endothelial dysfunction, oxidative stress, inflammation, autonomic imbalance, thrombosis, dehydration, and circadian disruption. “Contextual pathways” refer to built, social, occupational, and health-system conditions that shape exposure, vulnerability, and access to prevention or acute stroke care. The figure does not imply equal effect size, equal quality of evidence, or direct causality across all domains; current evidence is strongest for air pollution, wildfire smoke, and thermal stress. Acute environmental disruptions and disasters may influence stroke risk both directly and indirectly, including through intensified exposures and disrupted access to care. **Abbreviations**: PM₂.₅, fine particulate matter with aerodynamic diameter ≤ 2.5 μm; NO₂, nitrogen dioxide

Across these domains, proposed biological and health-system pathways related to increase stroke risk include endothelial dysfunction, oxidative stress, inflammation, autonomic imbalance, hypercoagulability and thrombosis, dehydration, circadian disruption, psychosocial stress responses, and impaired access to care [3, 7–16]. Table 1 summarizes representative recent studies examining environmental exposures and stroke risk. Table 2 outlines the major exposure domains, their proposed biologic mechanisms, and associated stroke outcomes. Figure 1 shows how environmental exposures may increase stroke risk through shared biologic and contextual pathways.

Throughout, the most useful question is not whether every environmental exposure independently “causes” stroke, but which exposures are sufficiently consistent, biologically plausible, and actionable to matter in contemporary stroke prevention and risk stratification. Moreover, the complex interplay of association of traditional risk factors with environmental, sociodemographic and geographic ones and wethere the share a causal relationship or not is paramount. This review focuses on the most clinically relevant and best-supported recent evidence on environmental exposures and stroke risk.

**Table 1:** Mapping of environmental exposure domains across representative recent stroke studies

Study	N*	Study Design	Air pollution /smoke	Temperature /heat	Noise / circadian /occupational	Built / social environment	Metals / toxicants	Natural Disasters
Niu et al. [3]	> 23 million	SR + MA	P	NR	NR	NR	NR	NR
Thacher et al. [17]	78,389 participants	Cohort Study	NR	NR	P	NR	NR	NR
Poulsen et al. [5]	94,256 stroke cases	Cohort Study	P	NR	P	P	NR	NR
Khadke et al. [2]	72,337 US census tracts	National ecological census-tract study	P	NR	NR	P	P	P
Qu et al. [9]	204 countries/territories	Ecological burden study	NR	P	NR	NR	NR	NR
Lu et al. [18]	13,696 participants	Cohort Study	NR	NR	NR	P	NR	NR
Jin et al. [12]	700,742 subjects	SR + MA	NR	NR	P	NR	NR	NR
Pershagen et al. [6]	> 8.4 million individuals	SR + MA	NR	NR	P	NR	NR	NR
Yin et al. [8]	329,876 ischemic-stroke admissions	Ecological time-series study	NR	P	NR	NR	NR	NR
Chen et al. [19]	204 countries/territories	Ecological burden study	NR	NR	NR	NR	P	NR
Hao et al. [4]	~ 25 million	Cohort Study	P	NR	NR	NR	NR	NR
Valente et al. [16]	114 reviews included	Umbrella review	P	P	NR	NR	NR	P
Vijaykumar et al. [20]	897 respondents across 4 countries	Cross-sectional Study	NR	NR	NR	NR	NR	P
Gopang et al. [15]	19 studies	SR + MA	NR	NR	NR	NR	P	NR
Meijer et al. [21]	3,019,069 participants	Cohort Study	NR	NR	NR	P	NR	NR
Wu et al. [22]	81,368 participants	Cohort Study	NR	NR	NR	P	NR	NR
Windred et al. [11]	88,905 participants	Cohort Study	NR	NR	P	NR	NR	NR
Pega et al. [13]	194 countries/territories	Systematic burden analysis/modeling study	NR	NR	P	NR	NR	NR
Fransson et al. [14]	196,380 participants	MA	NR	NR	P	NR	NR	NR
Chu et al. [7]	1,051,267 adults with stroke	Time-stratified case-crossover study	NR	P	NR	NR	NR	NR
Negev et al. [23]	22,269 first stroke events + 8,728 first TIAs	Time-stratified case-crossover study	NR	P	NR	NR	NR	NR
Fan et al. [10]	159 studies	SR + MA	NR	P	NR	NR	NR	NR
Saad et al. [24]	191 studies	Scientific statement	P	P	NR	NR	NR	P

Abbreviations: *SR* + *MA* systematic review and meta-analysis, *MA* meta-analysis, *NR* not reported or not addressed as a study domain, *P* present/addressed in the study, *TIA* transient ischemic attack

N* refers to the analytic unit reported by each study and may indicate participants, stroke cases, admissions, census tracts, countries/territories, reviews, or included studies

**P** indicates that the environmental exposure domain was represented or evaluated in the study. **NR** indicates that the domain was not reported or was not a focus of that study

**Table 2:** Summary of major environmental exposure domains, mechanisms of stroke, and stroke outcomes

Exposure domain	Mechanistic pathways to stroke	Stroke incidence / long-term risk	Stroke admission / short-term trigger	Mortality, burden, and temporal trends
Air pollution and combustion-related exposures	Inhaled particulates and combustion gases may promote oxidative stress, systemic inflammation, endothelial dysfunction, autonomic imbalance, hypercoagulability, blood-pressure lability, and accelerated atherosclerosis	Ambient air pollution, especially PM₂.₅, remains one of the most consistent long-term environmental stroke-risk exposures; newer data suggest wildfire-smoke PM₂.₅ may be an important cumulative combustion-related risk factor.	Short-term evidence supports air pollution as an acute stroke trigger, with higher admission risk reported for several particulate and gaseous pollutants.	Air pollution contributes to stroke mortality and population burden; wildfire smoke adds a growing contemporary burden signal, especially where smoke exposure is recurrent.
Thermal environment and climate-related stroke risk	Thermal stress may cause dehydration, hemoconcentration, blood-pressure variability, autonomic activation, endothelial dysfunction, and prothrombotic change; humidity amplifies heat load, while cold may increase vascular tone and blood viscosity.	Nonoptimal temperature is linked to stroke risk, with recent evidence strongest for acute heat effects and population burden rather than classic long-term incidence.	Heat is one of the clearest immediate environmental stroke triggers, especially under humid, high-heat-index, or heatwave conditions; cold remains relevant but is less consistently isolated as a short-term admission trigger.	Temperature contributes substantially to stroke mortality and DALY; low temperature still accounts for most temperature-attributable burden, while high-temperature burden is increasing rapidly, particularly in older and socially vulnerable populations.
Sleep-circadian disruption, noise exposure, and occupational factors	Noise, sleep fragmentation, circadian misalignment, long working hours, job strain, and occupational noise may converge through sympathetic activation, stress-axis activation, impaired blood-pressure regulation, inflammation, metabolic strain, endothelial injury, and stress-related behavioral changes.	Road-traffic noise, artificial light at night, shift/night work, long working hours, and job strain are best understood as chronic or cumulative stroke-risk contributors. The strongest occupational burden signal is for long working hours, while job strain appears more subtype-specific for ischemic stroke; occupational noise evidence is weaker and less consistent.	Short-term admission data are limited. These exposures are usually studied as chronic or cumulative stressors, although recent high occupational noise exposure may be more relevant than remote exposure.	Long working hours have the clearest evidence for stroke mortality and DALY burden, whereas circadian, noise, and psychosocial workplace exposures currently have stronger evidence for incidence or risk than for formal burden-trend estimates.
Built and social environment, and environmental inequity	Greenness, walkability, housing, neighborhood safety, food access, transportation, social connection, and socioeconomic conditions may shape physical activity, pollution and noise exposure, heat burden, chronic stress, sleep, healthcare access, and long-term vascular risk-factor control.	Greenness appears potentially protective, low walkability tracks with poorer cardiovascular health, and low socioeconomic status, deprivation, loneliness, and social isolation are linked to higher long-term stroke risk or vulnerability.	These are not classic immediate stroke triggers, but they influence acute presentation and care access, including delayed arrival, reduced access to stroke-unit care, and barriers to prevention in disadvantaged groups.	Persistent socioeconomic gradients remain evident for stroke disability and mortality, whereas greenness, walkability, and social-isolation studies currently contribute more to long-term risk framing than to formal stroke burden-trend estimates.
Waterborne, metals, and other toxicants	Toxicants may contribute through oxidative stress, endothelial toxicity, vascular inflammation, arterial stiffness, atherosclerosis progression, and prothrombotic change; microplastics and nanoplastics are emerging candidates with plausible inflammatory and plaque-related effects.	Arsenic shows positive but heterogeneous associations with stroke incidence, lead remains linked to persistent ischemic-stroke burden, and microplastics/nanoplastics are an emerging vascular signal associated with composite cardiovascular outcomes that include stroke.	Short-term stroke-admission evidence is limited; these toxicant exposures are studied mainly as chronic contributors rather than immediate triggers.	Lead remains a persistent contributor to ischemic-stroke deaths and DALY in recent burden analyses, arsenic has more heterogeneous mortality evidence, and microplastics/nanoplastics remain too newly studied for firm mortality-burden or temporal-trend conclusions.
Acute environmental disruptions and disasters	Disasters may combine pollutant and thermal exposure with acute stress, dehydration, displacement, medication interruption, communication failures, and delays in emergency stroke pathways.	Evidence is mainly observational and indirect, but repeated wildfire, heat, storm, flood, and displacement exposures may increase cerebrovascular vulnerability through cumulative exposure, stress, and disrupted continuity of care.	Wildfires, heat emergencies, hurricanes, floods, power outages, and evacuations can increase healthcare demand and delay emergency response, with medication interruption, transport disruption, and information gaps acting as major modifiers.	Increasing frequency and intensity of climate-related extremes may heighten stroke-relevant vulnerability, although stroke-specific burden quantification remains incomplete for several disaster types.

Abbreviations: *BP* blood pressure, *DALY* disability-adjusted life-year, *PM* particulate matter, *PM*₂.₅ fine particulate matter with aerodynamic diameter ≤ 2.5 μm, *SES* socioeconomic status

### Air Pollution and Combustion-related Exposures

Ambient air pollution remains one of the most consistent environmental contributors to stroke risk; perhaps in-part due to the ability to measure it and capture the data across large dtasets. Fine particulate matter with an aerodynamic diameter ≤ 2.5 μm (PM₂.₅) is especially important because it can penetrate deeply into the respiratory tract and trigger systemic vascular effects. Fossil-fuel combustion remains a major source of ambient PM₂.₅, alongside traffic emissions, industrial activity, residential combustion, dust, and secondary aerosols formed through atmospheric reactions. In an updated systematic review and meta-analysis, Niu et al. found that PM₂.₅ was associated with increased stroke hospital admission, incidence, and mortality, while particulate matter with aerodynamic diameter ≤ 10 μm (PM₁₀), nitrogen dioxide (NO₂), sulfur dioxide (SO₂), carbon monoxide (CO), and ozone (O₃) showed positive associations with selected stroke outcomes, particularly hospital admission and mortality [3]. Earlier cohort evidence also linked long-term PM₂.₅ exposure with increased cardiovascular and cerebrovascular events, supporting the vascular relevance of chronic particulate exposure [21].

Wildfire smoke represents a distinct and increasingly important combustion-related PM₂.₅ exposure. Unlike background urban or regional PM₂.₅, wildfire-smoke PM₂.₅ is episodic, geographically mobile, and capable of producing repeated multi-day surges in particulate exposure, including in regions with otherwise modest average pollution levels. In a nationwide U.S. cohort of approximately 25 million Medicare beneficiaries, Hao et al. reported that each 1 µg/m³ increase in 3-year average wildfire-smoke PM₂.₅ was associated with a 1.3% higher risk of incident stroke, with stronger per-unit associations than those observed for non-smoke PM₂.₅ [4]. These findings suggest that recurrent wildfire-smoke exposure may contribute to cumulative cerebrovascular risk rather than acting only as an isolated acute exposure.

The broader pollution literature also indicates that particulate exposure should not be considered in isolation. In a nationwide Danish cohort with 94,256 stroke cases, Poulsen et al. found that PM₂.₅ remained independently associated with stroke after adjustment for road-traffic noise and residential green space, while apparent associations with green-space measures were attenuated after accounting for co-exposures [5]. This supports the importance of multi-exposure approaches when evaluating environmental stroke risk, particularly in urban and transportation-related environments where air pollution, noise, and neighborhood conditions often co-occur [5].

These associations are biologically plausible. Proposed pathways linking PM₂.₅ and related pollutants with stroke include oxidative stress, systemic inflammation, endothelial dysfunction, autonomic imbalance, thrombosis, increased blood pressure, vascular injury, and accelerated atherosclerosis, which are relevant to plaque instability and cerebral ischemia [3, 21]. Overall, the most plausible contemporary message is that long-term ambient PM₂.₅ remains the clearest pollution-related stroke signal, while wildfire-smoke PM₂.₅ should be treated as a distinct and increasingly important combustion-related exposure [3–5].

### Thermal Environment and Climate-related Stroke Risk

Among climate-related exposures, heat has emerged as a potential acute stroke trigger. In a large U.S. case-crossover study of adults aged 18–64 years, stroke risk increased at higher daily mean temperatures, with an odds ratio of 1.30 at the 95th temperature percentile compared with the minimum morbidity temperature [7]. In a warm-season Israeli National Stroke Registry case-crossover study, higher ambient temperature, relative humidity, and heat index were each associated with increased stroke risk: 90 °F (32 °C) versus 81 °F (27 °C) was associated with higher total stroke risk, 90% versus 70% relative humidity showed the strongest association two days before stroke onset, and a heat index of 100 °F versus 80 °F was associated with approximately 40% higher total stroke risk on the event day [22]. In Hunan Province, China, heatwaves and heatwave characteristics were associated with increased ischemic stroke hospital admissions, with higher relative humidity during heatwaves further increasing admission risk [8]. Together, these findings support high ambient temperature and humid heat stress as clinically relevant short-term stroke exposures [7, 8, 22].

Temperature-related stroke risk is biologically plausible. Heat may promote dehydration, hemoconcentration, blood-pressure instability, endothelial stress, and prothrombotic changes, while humidity increases physiologic heat load by impairing evaporative cooling [7–9].

Global burden data suggest that temperature-attributable stroke burden is greater in lower Socio-demographic Index (SDI) regions, potentially reflecting differences in medical access, cooling resources, poverty, and heat exposure. Broader environmental-justice evidence also links cumulative social and environmental disadvantage with higher stroke prevalence [2, 9]. These inequities are clinically relevant because access to air conditioning, cooling centers or other safe cooling spaces, reliable electricity, transportation, and timely medical care may determine whether extreme heat becomes a manageable exposure or a stroke-relevant emergency [2, 16].

Cold exposure also remains relevant, although it appears to operate through a different pattern. Meta-analytic evidence links lower temperature and cold spells with increased cardiovascular morbidity and mortality, with supportive evidence for stroke-related mortality [10]. Global burden analyses further indicate that low temperature currently accounts for most temperature-attributable stroke deaths and disability-adjusted life-years (DALY) worldwide, while the burden attributable to high temperature is rising rapidly [9]. A recent World Stroke Organization scientific statement similarly concluded that cold exposure, temperature variability, and extreme thermal events are among the most consistently associated climate-related stroke exposures, while heat-related effects appear to be increasing over time. The statement also highlighted possible contributions from humidity shifts, barometric-pressure variability, and compound weather events, particularly in older adults and low- and middle-income settings [18].

These findings support incorporating stroke-relevant outcomes into heat-health preparedness, including heatwave warnings, cooling centers, and other accessible cooling strategies, backup power planning, transportation support, medication continuity, and targeted outreach to socially vulnerable populations [2, 8, 9, 16]. Clinicians should be aware that nonoptimal temperature as a practical environmental exposure domain may have relevance, rather than as background context alone [7–10].

### Acute Environmental Disruptions and Disasters

Disasters deserve separate attention because they can intensify hazardous environmental exposures while simultaneously disrupting the systems needed for prevention, emergency response, and continuity of care. As discussed above, wildfire-smoke PM₂.₅ is associated with incident stroke, making wildfires an important combustion-related cerebrovascular exposure [4]. Floods, storms, heatwaves, cold spells, and wildfires shows effects on prehospital response, emergency departments, hospitals, primary care, pharmacies, and other health-system functions [16]. Heat emergencies can increase ambulance dispatches, emergency visits, and hospitalizations, while also straining transportation, electricity, medication storage, and clinical capacity [16]. Storms and floods can damage facilities, block transport routes, interrupt medications and follow-up care, and disrupt communication systems needed for timely protective action [16, 17]. Recent flood and hurricane case studies further show that disasters create “information voids” across information quantity, quality, trusted sources, and accessible communication channels, which may impair evacuation, access to health services, and emergency decision-making [17]. Thus, environmental stroke risk during disasters depends not only on exposure intensity, but also on community and health-system resilience, including reliable communication, evacuation planning, backup power, transportation access, and medication-continuity measures [16, 17].

### Sleep-circadian Disruption, Noise Exposure, and Occupational Factors

Several environmental and occupational exposures including road-traffic noise, artificial light at night, shift/night work, long working hours, job strain, and occupational noise, may converge on overlapping stroke-relevant pathways. These include sleep fragmentation, circadian misalignment, sympathetic activation, impaired blood-pressure regulation, metabolic dysregulation, endothelial injury, inflammation, and stress-related behavioral changes. Compared with the evidence for air pollution and temperature-related exposures, associations for these sleep, circadian, noise, and occupational domains are generally more modest, heterogeneous, or outcome-specific. Given their relevance, chronicity, and co-occurence in modern urban and working environments these associations have been studied [6, 11–14, 19].

A recent systematic review and meta-analysis of longitudinal studies found that long-term road-traffic noise exposure was associated with a modest increase in incident stroke risk per 10 dB increase [6]. However, evidence differs by noise source and exposure context. In a pooled Scandinavian cohort study of 78,389 participants, baseline occupational noise exposure was not clearly associated with overall stroke after full adjustment, although ischemic stroke estimates were modestly elevated, and time-varying analyses suggested that recent high occupational noise exposure may be more relevant than remote exposure [19]. Taken together, these findings support a cautious interpretation that chronic noise exposure may contribute to stroke risk, but the evidence is less consistent than for major particulate or thermal exposures [6, 19].

Artificial light at night has emerged as a related circadian exposure. In a large UK Biobank analysis using personal light-sensor data, participants with the brightest nighttime light exposure had a 28% higher risk of incident stroke than those with the darkest nights [11]. The association remained robust after adjustment for socioeconomic and lifestyle factors, although it was attenuated in some sensitivity analyses that additionally accounted for short sleep and lipid-related factors [11]. A plausible interpretation therefore is not that night light alone causes stroke, but that it may mark or contribute to a broader pattern of circadian disruption and urban environmental stress contributing to this risk.

A major limitation across these exposure domains is measurement. Road-traffic noise is often modeled from residential address, occupational noise may rely on job-exposure matrices or baseline workplace estimates, and shift-work or job-strain measures are frequently self-reported or assessed at a single time point. These approaches may miss indoor conditions, commuting exposures, hearing protection, time spent away from home or work, changes in work schedules, sleep timing, and individual susceptibility. The use of personal light sensors in the UK Biobank light-at-night study illustrates how wearable and near-person exposure measurement may improve future research [11]. Future studies combining personal light exposure, sleep/activity monitoring, noise dosimetry, geolocation, heart-rate or autonomic measures, and environmental sensors may help clarify whether stroke risk is driven by exposure intensity, timing, duration, or clustering with other social and occupational stressors [6, 11, 12, 19].

Occupational rhythms and psychosocial work exposures reinforce this pattern. Recent meta-analytic evidence suggests that shift and night work are associated with a modest increase in stroke risk [12]. Long working hours show stronger burden-related evidence: working 55 h or more per week has been associated with increased stroke risk and contributes to global stroke mortality and DALY burden [13]. Job strain appears more subtype-specific; in an individual-participant data meta-analysis, job strain was associated with higher ischemic stroke risk, whereas associations with overall stroke and hemorrhagic stroke were not clearly significant [14].

The clinical implication is less about screening for one exposure at a time than about recognizing exposure bundles. An individual with poor sleep, heavy commuting noise, bright nights, rotating shifts, overtime work, job strain, and poorly controlled hypertension may carry a stroke risk profile that is not fully captured by conventional vascular history alone. Future research should therefore favor multi-exposure models, improved personal exposure measurement, and intervention studies focused on schedule design, sleep protection, noise reduction, and healthier working conditions.

### Built and Social Environment and Environmental Inequity

The built environment and social context may influence stroke risk through multiple, overlapping pathways rather than through single exposures in isolation. Neighborhood greenness, walkability, transportation conditions, and broader social disadvantage can shape physical activity, stress, cardiometabolic risk, environmental exposure burden, and access to care. Because these features often cluster within places, they are better understood as exposure patterns embedded within communities than as fully separable risk factors.

“Greenness” remains one of the more plausible protective exposures in this domain. In a prospective cohort of 13,696 middle-aged and older Chinese adults, lower green-space availability was associated with a higher risk of new-onset stroke, and the relationship appeared nonlinear, with the steepest benefit occurring at lower levels of greenness before flattening beyond an estimated threshold of approximately 10.6 m² of park green area per person [20]. This pattern is consistent with the idea that relatively small increases in usable green space may matter most where greenness is scarce.

Walkability likely operates through related mechanisms. In a nationwide Dutch cohort of 3,019,069 adults, long-term exposure to low-walkability neighborhoods was associated with a higher risk of cardiovascular disease overall, while stroke-specific estimates were directionally similar but weaker and not statistically significant [25]. The broader implication is still important: environments that make routine walking difficult may promote inactivity and worsen cardiometabolic risk over time, making built-form decisions relevant to vascular health.

The social environment magnifies these patterns. A recent review concluded that lower socioeconomic status is consistently associated with higher stroke risk, lower-quality care, greater disability, and higher mortality, with much of this inequality operating through poorer control of conventional vascular risk factors and persistent differences in access to care [1]. These gradients are unlikely to be explained by any single mechanism. Rather, socioeconomic disadvantage often coexists with greater exposure to environmental hazards and reduced access to preventive and acute stroke care. In a UK Biobank analysis, lower levels of loneliness and social isolation were associated with lower stroke risk among people with diabetes over 12 years of follow-up, with stronger associations in men than in women [23]. These findings support viewing social disconnection not simply as a psychosocial correlate but as part of the broader context in which stroke risk may accumulate, particularly in high-risk cardiometabolic populations.

This is also the point at which environmental medicine and stroke equity most clearly converge. In a national US census-tract analysis, communities with the highest cumulative environmental vulnerability had higher stroke prevalence and a greater burden of adverse cardiovascular conditions than communities with the lowest vulnerability [2]. For clinicians, this suggests that environmental history may include practical contextual questions: whether a patient lives in a neighborhood with low greenness, poor walkability, heavy traffic exposure, weak transportation access, or other signs of cumulative environmental disadvantage. Such questions may complement standard vascular risk assessment, and can help explain why conventional prevention may underperform in structurally disadvantaged settings.

### Waterborne, Metals, and Other Toxicants

Compared with air pollution and temperature, recent stroke-related evidence for waterborne and other toxicant exposures remains narrower and more heterogeneous. Among these exposures, lead remains the most defensible legacy toxicant: contemporary global burden analyses attribute a measurable ischemic stroke burden to lead exposure, despite declining average exposure levels in some high-SDI settings, underscoring persistent risk from sources such as dust, old paint, drinking water, plumbing, food, and occupational or industrial exposures [26]. Arsenic also remains relevant within a waterborne toxicant framework, although the evidence is less uniform. A recent systematic review found a positive association between urinary arsenic and stroke incidence, while drinking-water arsenic showed a more suggestive and methodologically heterogeneous association with stroke incidence [15].

Microplastics and nanoplastics are best regarded as emerging vascular toxicants rather than established stroke risk factors. In patients undergoing carotid endarterectomy, microplastics and nanoplastics were detected within carotid plaque, and plaque positivity was associated with a higher risk of a composite endpoint of myocardial infarction, stroke, or death, along with a more inflammatory plaque phenotype [24].

Future work should move beyond single-toxicant associations toward better exposure characterization, biomonitoring, and source attribution. For legacy metals such as lead and arsenic, priorities include identifying high-risk communities, clarifying dose–response relationships at low-to-moderate exposure levels, and linking environmental measurements with individual-level stroke outcomes. For emerging contaminants such as microplastics and nanoplastics, standardized methods for detection, quantification, and tissue characterization are needed before their role in stroke can be defined. Studies that combine environmental sampling, blood or urine biomarkers, vascular imaging, inflammatory markers, and longitudinal stroke follow-up will be particularly important for determining whether these toxicants are causal contributors, markers of broader environmental burden, or both.

## Conclusions

Environmental factors are an emerging risk linked to stroke and worthy of future research. Recent literature supports a hierarchy of evidence in which air pollution, wildfire smoke, and nonoptimal temperature—particularly extreme heat—show the most consistent and possibly, actionable associations with stroke risk and burden. Circadian disruption, noise, and workplace stressors appear to contribute more modestly but plausibly, particularly when they cluster with hypertension, sleep disruption, and psychosocial stress. Built and social environments are harder to succinctly quantify, yet may be among the most important real-world determinants because they concentrate multiple harmful exposures while constraining access to prevention, emergency response, and acute stroke care.

For clinicians, the practical implication is not to replace traditional vascular risk assessment, but to widen it. Environmental context helps explain why risk accumulates, why prevention performs unevenly across populations, and where policy and systems-level action may matter. For researchers, the next step is to move beyond single-exposure models toward cumulative exposome frameworks that integrate new data : geospatial exposure, wearable technologies, electronic health records, and machine-learning approaches. Such tools may improve measurement of dynamic exposures such as light at night, sleep, noise, heat, smoke, mobility, and disaster-related disruptions, while also helping identify vulnerable populations and exposure clusters that are missed by traditional epidemiologic designs.

The stroke exposome should also remain open to emerging hazards. Microplastics and nanoplastics, for example, have been detected in human carotid plaque and are associated with subsequent cardiovascular events in a high-risk vascular population, but they are not yet established independent stroke risk factors. Their emergence underscores a broader point: environmental stroke prevention must remain adaptive as new contaminants, climate-related hazards, and built-environment risks are identified. For health systems, the immediate opportunity is to integrate stroke prevention with climate preparedness by advancing cleaner-air policies, safer workplaces, resilient communication and transport systems, and more equitable neighborhood design. This direction aligns with recent World Stroke Organization recommendations advocating climate-aware stroke prevention, early-warning mechanisms, patient education, resilient health-system planning, and focused protection of populations with the least adaptive capacity.

## Key References

- Hao H, Xu K, Zhang D, et al. Long-term exposure to wildfire smoke particulate matter and incident stroke: a US nationwide study. Eur Heart J. 2026. doi:10.1093/eurheartj/ehaf875
  - ○ A large nationwide cohort study showing that long-term wildfire-smoke PM₂.₅ exposure is associated with incident stroke, supporting the manuscript’s emphasis on wildfire smoke as a distinct and increasingly important cerebrovascular exposure.
- Poulsen AH, Sørensen M, Hvidtfeldt UA, et al. Concomitant exposure to air pollution, green space, and noise and risk of stroke: a cohort study from Denmark. Lancet Reg Health Eur. 2023;31:100655. doi:10.1016/j.lanepe.2023.100655
  - ○ An important multi-exposure cohort study showing that stroke risk should be considered in the context of clustered environmental exposures rather than isolated hazards, closely matching the manuscript’s exposome framework.
- Negev M, Paz S, Vered S, Kloog I, Weinstein G. High ambient temperature, humidity, heat index, and stroke risk in a Mediterranean region. Int J Stroke. 2026. doi:10.1177/17474930261429880
  - ○ Direct evidence that heat, humidity, and heat index are clinically relevant short-term stroke exposures, reinforcing the manuscript’s focus on thermal stress and heat-humidity metrics as actionable stroke triggers.
- Khadke S, Kumar A, Al-Kindi S, et al. Association of environmental injustice and cardiovascular diseases and risk factors in the United States. J Am Heart Assoc. 2024;13(7):e033428. doi:10.1161/JAHA.123.033428
  - ○ A strong environmental-justice study linking cumulative environmental disadvantage with higher stroke prevalence and worse cardiovascular risk profiles, supporting the manuscript’s emphasis on unequal burdens and structural inequity.
- Saad A, Khan M, Estol C, et al. Stroke and climate change: A World Stroke Organization scientific statement. Int J Stroke. Published online March 15, 2026. doi:10.1177/17474930261436535
  - ○ An authoritative scientific statement that synthesizes the climate-stroke literature and supports the manuscript’s broader message that stroke prevention and stroke systems of care should incorporate climate awareness, preparedness, and protection of vulnerable populations.

## Data Availability

No datasets were generated or analysed during the current study.
